# Social Network, Cognition and Participation in Rural Health Governance

**DOI:** 10.3390/ijerph19052862

**Published:** 2022-03-01

**Authors:** Jiayi Tang, Haibo Ruan, Chao Wang, Wendong Xu, Changgui Li, Xuan Dong

**Affiliations:** 1 School of International Relations & Public Affairs, Fudan University, Shanghai 200433, China; tangjy21@m.fudan.edu.cn; 2 Institute of China Rural Studies, Central China Normal University, Wuhan 430079, China; hslijie@ccnu.edu.cn; 3 School of Public Policy & Management (School of Emergency Management), China University of Mining and Technology, Xuzhou 221116, China; wangchaoccnu@163.com (C.W.); ts20090007a31@cumt.edu.cn (X.D.); 4 School of Foreign Studies, China University of Mining and Technology, Xuzhou 221116, China; xwdspace@163.com

**Keywords:** social network, rural resident cognition, rural health governance, introverted communication, responsibility awareness

## Abstract

Rural health governance is an important part of low-carbon green life, which is also related to the sustainable development and population health project in rural areas. Based on the survey data of 2343 rural residents in China, this study adopted a binary logistic regression model to explore the effects of rural residents’ social network and cognition on their participation in rural health governance. The research results show that only less than 30% of the respondents participated in rural health governance, and the proportion of rural resident participating is not high. Both their social network and cognition have a significant impact on their participation in rural health governance. Introverted communication helps strengthen the connection between rural residents, form the network and structure of rural social communication, build emotional links and common interests, and form a common cultural understanding paradigm and action framework. The extraverted communication means that rural residents gradually break away from the social network of acquaintances, which is not conducive to building a rural community. Rural residents’ understanding of behavior begins to deviate from rural culture, customs and emotional values, and the binding force of traditional culture is reduced, making it difficult to motivate them to participate in rural public life. Policy cognition can improve rural residents’ recognition of the value and significance of health governance. Responsibility awareness is the internal driving force for rural residents to participate in health governance, which can also reduce the governance cost of rural managers. Based on this, increasing rural residents’ introverted communication and cultivating their sense of responsibility are key to promoting their participation in rural health governance.

## 1. Introduction

Entering the 21st century, the expectations from the international community for a higher-quality living environment have been increasing day by day, and the concept of sustainable development has been advocated worldwide. At the same time, China also attaches great importance to environmental protection and vigorously promotes the construction of an ecological civilization. However, in the vast rural areas of China, there is still a problem of sacrificing the environment in exchange for economic development. The implementation of reform and opening policies has brought unprecedented opportunities to rural areas where the market has mobilized all human and material resources, effectively boosting the rural economy in China. According to the National Bureau of Statistics of China, the per capita income of rural residents increased from CNY 133 in 1978 to CNY 17,131 in 2020. However, the development of the rural economy and the increase in rural residents’ income levels have not brought about the improvement of rural environmental governance. Rural household waste, construction waste, solid building waste, etc. are randomly stacked, and the building of environmental governance infrastructure is inadequate, resulting in problems including “garbage blown away by wind, sewage evaporated to air”, “modernized indoor, while dirty and messy outdoor” [1]. Rural residents’ life quality has been reduced as a result of certain environment damages caused by rural economic development [2].

The rapid development of the rural economy and the imperfect governance of the public health environment are accompanied by changes in the social interactions of rural residents. First, the improvement of traffic conditions has expanded the scope of the social interaction of rural residents and is no longer limited to the scope of rural areas. This increases the possibility of rural residents to communicate between urban and rural areas in space, and the construction of urban and rural roads also reduces the cost of rural residents’ communication [3]. The second is the change in the type of social interaction among rural residents. Social interaction in the rural community is based on communication between acquaintances, with common customs and cultural concepts, and the understanding of behavioral paradigms is homogeneous. Communication between villagers incorporates the cultural and emotional factors of fellow villagers, making it possible to help each other, while the social communication expanded by rural residents is more of a market transaction nature and follows the principles of freedom, voluntariness, and equality. Social interaction outside the countryside is based on the trading rules of the capital and market. The third is the change in the way of social interaction of rural residents. The popularity of the Internet has provided more options for communication. Online communication and interaction integrate language, video, action, etc., so that members can communicate in a variety of ways. Some researchers pointed out that the promotion of the Internet in rural areas can improve residents’ willingness to participate in rural environmental governance [4]. Thus, has the transformation of rural residents’ social interaction changed their participation in rural health governance?

In February 2018, Chinese government formulated a 3-year initiative specifically to improve rural residential environments in order to promote rural residential living qualities as well as rural health governance. The initiative focuses on four aspects of rural environmental governance, including rural domestic waste management, toilet excrement treatment, domestic sewage management, and village appearance management. After three years of rectification actions, the rural living environment has continued to be improved, and the appearance of the village has been significantly improved. Looking back at the 3-year plan on the improvement of the rural residential environment from 2018 to 2020, it can be found that rural residents are not so active in participating in the improvement of the residential environment sanitation. Rural residents’ subjectivity is insufficient, which in turn affects their satisfaction with the improvement of a rural residential environment [5]. In the context of ongoing urbanization, a large number of rural people have migrated to cities. The resulting hollowing and aging of the countryside brings difficulties to collective action for environmental governance. Changes in the way and scope of social interaction among rural residents also pose challenges to the realization of rural public behavior [6]. The social contacts of acquaintances that already exist in the countryside are disintegrating. Original mutual help, public welfare behavior, and altruism are decreasing. “Rational small farmers” and “interest-oriented small farmers” are more prominent. The “environment” is a rural public good with positive externalities. Reducing the existence of the tragedy of the commons, increasing the effective provision of environmental governance, and stimulating the subjectivity and enthusiasm of rural residents’ participation are all very necessary [7]. Therefore, studying the impact of social interaction on rural residents’ participation in rural health governance is of great significance for the provision of rural environmental products.

The low enthusiasm of rural residents to participate may influence the general attitude of the government to dominate public affairs [8]. Based on this, studies have been conducted to explore the influencing factors of rural residents’ participation in rural health governance. Xia Zhao et al. believed that rural sanitation conditions, housing conditions, infrastructure conditions, rural residents’ economic affordability, and rural public services affect the governance of rural residents [9]. Iwona Pomianek found that changes in rural population and rural labor structure influenced rural environmental governance [10]. Yue Shen et al. emphasized the influence of the layout of sanitation and environmental facilities and geographic location on rural residents’ participation behavior. If the location of environmental facilities construction can show common interest needs, the formation of social network relationships among rural residents can promote participation behavior [11]. Jane Mills et al. conducted a study on the motivation of rural residents to participate and found that rural residents would take environmental protection behaviors in order to obtain agricultural subsidies [12]. Chen Qing et al. discussed the impact of rural residents’ awareness of environmental protection on their participation in their behavior. The awareness of environmental issues, environmental pollution tolerance and environmental attitudes can all actively promote rural residents’ environmental protection behaviors [13]. Yu Chen’s research found that rural health governance are rural public goods, which are non-exclusive and non-competitive. Rational rural residents will choose free-riding behavior and can enjoy the results of rural environmental governance without participating in environmental governance [14]. Zhenghua Deng’s further classification research found that rural residents enjoying superior economic conditions are more likely to choose free-riding behavior [15], because rural residents with higher economic incomes show that their rational thinking is more advantageous. Satola, Lukasz et al. discussed the influence of a local government’s financial autonomy on governance behaviors [16], finding that local debt [17], financial input [18], and stable rural funding sources [19] are important factors for the sustainability of environmental governance behavior. In addition, rural residents’ income [20], educational level, age [21] and sex [15] have been proven to have remarkable influence on their participation in environmental governance.

The studies mentioned above have played positive roles in promoting rural residents’ participation in rural health governance. However, these studies have not highlighted the special background of Chinese rural areas, i.e., the social network formed in the society of acquaintances. China’s rural areas are in the process of urbanization, and rural residents’ social interactions are also transforming. From a sociological point of view, Mr. Shuming Liang proposes to explain rural society as being “relationship-based”, thereby denying rural residents’ the rational choice of being “individual-based” [22]. Xiaotong Fei believes that rural society is based on a “Pattern of Difference Sequence” of a series of relationships [23]. The background of the traditional acquaintance society constitutes the basis of rural residents’ behavior choices, and their behavior choices are influenced by the rural social network and communication structure. Therefore, it is necessary to contextualize rural residents within the social background of acquaintances to study the influence of rural residents’ social interaction on their participation behavior. At the same time, the aforementioned research did not carry out classification exploration of rural residents’ cognition, and especially ignored the factors of rural residents’ cognition of responsibilities. Therefore, this research aimed to explore the impact of rural residents’ social network and cognition on rural residents’ participation in the rural health governance. At the same time, the previous studies did not classify and explore the environmental protection cognition of rural households, especially ignoring the factors of rural residents’ responsibility awareness, which is an important variable to maintain the sustainability of rural environmental governance [24]. Therefore, this study also aimed to explore the influence of social network and rural residents’ cognition on their participation in rural health governance.

## 2. Theoretical Backgrounds and Hypothesis Development

The governance of rural health has been a major approach in improving the outlook of rural villages which is directly related to the health of rural residents and the sustainable development of rural areas. At the same time, improving rural residential environment sanitation can solve the problem of inadequate development between rural economic development and green environmental protection. Rural health governance refers to the treatment of rural domestic waste, which includes solid waste, kitchen waste, paper, plastic, metal, glass, fabric, masonry, ash, pesticide packaging waste and hazardous waste such as discarded daily chemicals and expired medicines [25]. Rural health governance is an environmental governance behavior initiated by the government which transforms rural environmental governance into a government-implemented project and incorporates it into the scope of grassroots governance. Therefore, in this study, the so-called rural health governance refers especially to the governance action initiated by the government to deal with issues of rural public waste [26]. Rural residents are the core of the rural problem. The starting point of the rural health governance is for rural residents, and its realization path must rely on rural residents, give play to the main role of rural residents, and guide them to actively participate in rural health governance. Rural residents’ participation behavior should be understood based on the special social network of rural residents’ lives.

### 2.1. Social Network

The concept of social network comes from the theory of social capital and belongs to the category of social capital. In Putnam’s view, social capital is “the characteristics of social organizations such as trust, norms, and networks, which can increase the efficiency of society by promoting cooperative actions” [27]. At the same time, some scholars believe that social interaction belongs to the content of social network. It is precisely because of extensive social interactions that people form a relationship network, which in turn produces reciprocal norms and interpersonal trust. Therefore, a social network refers to the social activities in which people communicate with each other and carry out material and spiritual exchanges in a specific historical environment [28]. Social network activities help individuals contact each other, communicate and understand each other, and constitute the basis for individual survival, development, and participation. Through long-term social communication and interaction, rural residents’ activities shape their behavioral paradigm and cultural framework of mutual understanding. Individuals will integrate their behaviors into the familiar cultural definitions of the countryside so as to generate action trajectories that meet the expectations of others and the collective and thus avoid misconduct [29].

The traditional Chinese rural area is a society of acquaintances. The network of relationships composed of geographical, kinship, and industry connections is very rich. Frequent social interactions increase the feelings between rural residents. The regional location and the vulnerability of single households requires that rural residents form a close relationship. A community of interests or a community of villages which lays the foundation for human resources and emotions for participating in the rural health governance [30]. At the same time, with the advancement of reform and opening up and the market economy, the interaction between the countryside and the city has increased. Under the influence of the market mechanism, a large number of rural residents have left the countryside and entered the cities. On the one hand, rural areas are no longer closed. The social relations of acquaintances in rural areas are broken while the society of semi-acquaintances appears [31]; on the other hand, the rural residents’ social network is no longer confined to the interior of the village. Their social contacts change while rural–urban exchanges increase [32]. Based on the superposition of acquaintance society and semi-acquaintance society, this article divides rural residents’ social networks into two types: introverted communication and extroverted communication [33].

Introverted communication refers to the fact that the social interaction activities of rural residents are concentrated in the village, and their contacts are villagers from the same village. This type of communication is based on activities of kinship and acquaintances [34]. Since introverted communication occurs inside the village, its communication activities can easily form emotional connections, which is beneficial to the construction of rural communities and can find rural public interests. Introverted communication plays a role in three aspects: one is to establish a rich social network relationship in the village and to construct a rural social communication structure [35]; the other is to form an interest connection relationship based on the social communication network, and the interests of the villagers are interrelated, which produces a resonance effect. The mobilization of human resources has made preparations [36]; third, rich social interaction activities can form a village culture, which is especially conducive to the construction of a culture of integrity, which can alleviate the dilemma of collective action, increase rural residents’ consideration of public interests, and reduce the pursuit of personal interests [37]. Rural residents’ interactions with rural managers and other participants will increase their understanding of environmental protection knowledge and increase their attention to rural health governance [38].

Extroverted communication refers to the external communication activities of rural residents. The communication activities are beyond the scope of the village and constitute the communication carried out between the society of semi-acquaintances and the society of strangers [39]. The emergence of extroverted communication means that rural residents’ introverted interactions have decreased, and they have gradually entered the development of urbanization, separated from rural kinship-based and geographic-based interactions, and more of industry-based interactions [40]. Rural residents will acquire new values in these interactions, and gradually change their cognition from the countryside. Under certain circumstances, they will even oppose traditional countryside concepts, creating conflicts in terms of customs and culture [41]. Therefore, the emergence of extroverted communication will increase difficulties in the construction of rural communities. It is difficult for villagers to form interest relations since emotional construction is also in crisis, and concepts and consciousness are difficult to unify. Rural residents who go out will gradually divorce from rural public behaviors and become psychologically indifferent. Social relations will be reconstructed in cities [42]. The increase in extroverted communication among rural residents means that they acquire information, resources, technologies, and behavioral paradigms from outside the rural area. As a result, their own behavioral logic shifts to market logic and capital logic, lacking the connection of emotional logic.

The two following hypotheses are proposed based on the above analysis.

**Hypothesis** **1** **(H1).** *There is a significant positive correlation between introverted communication and rural residents’ participation in rural health governance.*

**Hypothesis** **2** **(H2).** *There is a significant negative correlation between extroverted communication and rural residents’ participation in rural health governance.*

### 2.2. Rural Residents’ Cognition

Rural residents’ cognition refers to the individual rural residents’ knowledge, judgment, and evaluation of the people, events, activities, and laws of the rural health governance [43]. Rural residents’ cognition represents the degree of rural residents’ understanding of the input or output of knowledge in the environmental governance system [44]. According to the theory of planned behavior, the individual’s subjective consciousness, attitude, and other psychological factors can explain the generation of individual behavior to a certain extent [45]. From a psychological point of view there is consistency between rural residents’ cognition and behavior, which can reflect the subject’s level of participation to a certain extent [46]. Psychological factors drive rural residents’ participation in terms of their behavior and cognition that contains basic personal values and are more likely to produce choices indicated by cognition [47]. At the same time, empirical research also shows that rural residents’ cognition has an impact on rural residents’ participation in environmental remediation. Research by Desheng Hu et al. found that the three dimensions of rural residents’ cognition, behavioral attitudes, perceived behavior control, and subjective norms all have a significant impact on rural residents’ participation in the improvement of residentials [48]. In this article, the rural residents’ knowledge is divided into two dimensions: one which is the rural residents’ policy cognition; and the other, which is the rural residents’ responsibility awareness.

Policy cognition is the individual’s cognition, judgment, and evaluation of the policy system and policy process. It is the psychological process of the interaction of the cognizant, the cognitive object and the situation, and it is the function, role, structure, and relationship of people to the policy system in addition to other cognitions. The more rural residents understand the policy, the more they can understand the significance of a government’s promotion of policy reform in their thinking and cognition, as well as the changes that the policy has brought to the countryside and can be consistent in value with the government [49]. The improvement of rural residents’ policy awareness can reduce the resistance to policy implementation, save the cost of policy implementation, and gain rural residents’ recognition. At the same time, the policy knowledge training carried out by the government can help improve the policy awareness level of rural residents, reduce the information asymmetry between the government and residents, and reduce the “information gap” between the two. From the perspective of the subjectivity of rural residents, disclosing environmental protection policies to them can increase their sense of ownership, increase their sense of responsibility, and incorporate their individual behaviors into the requirements of collective norms [50].

Responsibility awareness means that an individual believes that they or others should participate in the process of public affairs, regardless of whether these activities are worthy or costly [51]. The cognition of civic responsibility has two functions: restraining and stimulating: one is to restrain the bad behavior of individual citizens, reduce the harm to others to the collective, correctly handle the relationship between personal and collective interests, and correct the mentality of “free-riding” and “wait-and-see” among individuals; it can then encourage individual citizens to participate in collective activities, take behaviors that are beneficial to the individual and the collective, and transform the responsibilities entrusted by society into individual obligations [52]. Rural residents’ cognition of responsibility affects their division of responsibilities. The main bodies of rural health governance include the government, rural managers, and residents. How the three deal with the relationship of responsibilities and clarify their respective task scope and sense of responsibility will be conducive to efficient internal cooperation. Individual cognition is the basis for rural residents to participate in behavioral decision making and the internal motivation for individual participation [53].

Hypotheses 3 and 4 are proposed based on the above analysis.

**Hypothesis** ** 3** **(H3).** *There is a significant positive correlation between rural residents’ policy cognition and rural residents’ participation in rural health governance.*

**Hypothesis** **4** **(H4).** *There is a significant positive correlation between responsibility awareness and rural residents’ participation in rural health governance.*

**Figure 1:**
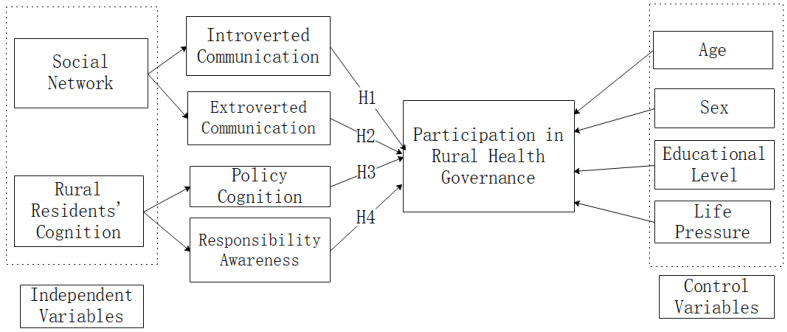
Conceptual model.

Figure 1 shows the conceptual model of the entire research hypothesis.

## 3. Methods

### 3.1. Data Sampling

In this study, questionnaire survey and semi-structured interview were used to analyze rural residents’ participation in rural health governance. The survey was conducted from January to February 2021. The questionnaire mainly includes individual characteristics, family information, social network, and rural residents’ cognition. The questionnaire was distributed in the form of offline interviews with researchers, and the respondents covered 25 provinces in mainland China except Hong Kong, Macao, Tibet, etc. In the selection of specific research sites, according to the population, regional distribution and other information, 6 cities (25 provinces × 6 cities) in each province were selected, and then 1 village was selected from each city (25 provinces × 6 cities × 1 village), and 15 rural residents were randomly selected from each village’s population register document. Considering the complexity of Chinese villages, some special villages are included in the survey, such as economically developed villages and villages at the forefront of reform. A total of 2460 questionnaires were sent out and 2343 were effectively received with an effective recovery of 95.24%, reaching the requirement of a 90% questionnaire efficiency. The research group randomly selected 10% of the respondents for a return visit to test the quality of the questionnaire data to ensure the authenticity of the survey data.

The semi-structured interview is a method used to extensively understand the internal influencing factors of rural residents’ participation in rural health governance. When illiteracy was encountered in the interview, a semi-structured interview was also used to complete the questionnaire survey. The investigator orally explained the survey content and purpose, asked the respondents according to the questionnaire questions, and the respondents answered orally, and the researcher recorded the content of the answers. At the same time, in order to obtain more sufficient first-hand information, the investigators also conducted informal interviews with grassroot government cadres, cadres of grassroot Party organizations, village managers to understand the overall situation, governance effects, and the existing problems of rural health governance. In addition, investigators also conducted informal interviews with rural residents to understand the situation of health governance in the village, so as to verify the information provided by village managers.

Due to the COVID-19 pandemic, the survey was not carried out nationwide but only in 25 provinces. In order to ensure the health and safety of personnel, the whole field research process strictly followed the COVID-19 Diagnosis and Treatment Protocol issued by the National Health Commission. Before the formal questionnaire survey, the investigator will briefly introduce the research and questionnaire survey to the respondents, and then the respondents will complete the questionnaire independently under the explanation of the investigator. Each survey took approximately 13–16 min, and all respondents were compensated for their time and help. All information obtained from the survey is anonymous and will be kept strictly confidential. Each respondent was required to fill out a written informed consent form to prove that participation in the study was entirely voluntary.

The sample characteristics of 2343 surveys are shown in Table 1. There were 1527 male respondents, accounting for 65.17%, and 816 female respondents, accounting for 34.83%; in terms of the age of the respondents, more respondents were 50–59 years old, 60 years old and above, accounting for 30.35% and 37.90%, respectively, which is consistent with the phenomenon of rural aging and hollowing out; in terms of the interviewees’ occupations, the respondents of agriculture and labor are the largest, accounting for 65% and 15.88%, respectively. In terms of marital status, there were 1976 married respondents, accounting for 84.34%; in terms of where the respondents lived, the proportions of respondents in the eastern, central, and western regions were 16.35%, 48.78%, and 34.87%, respectively. On the whole, the sample is in line with the actual situation in the rural areas, and it is quite real, and certain statistical analysis can be carried out.

### 3.2. Measurements

#### 3.2.1. Rural Residents’ Participation in Rural Health Governance

In this study, rural residents’ participation in rural health governance is regarded as a dependent variable, and the impact of social network and rural residents’ cognition on rural residents’ participation in the rural health governance was investigated. At the same time, the rural residents’ social network and cognition were divided into two dimensions to examine their impact effectiveness. The specific settings are shown in Table 2. This paper draws on the research methods of Tang Lin et al. [54], and sets the dependent variable as “Are you involved in rural health governance?” The answer is set as a binary variable, where “No” means no participation and “Yes” means participation, assigned values “0” and “1”, respectively. Rural residents’ participation behavior includes their choice of correct garbage disposal behavior and participation in rural garbage cleaning behavior. Their behaviors are only voluntary participation and exclude involuntary ones. At the same time, the health governance behavior of local organizations has not yet formed a fixed action mechanism or system, and most of them are temporary forms of participation.

#### 3.2.2. Social Network

According to the analysis of the theoretical foundation part, the social network in this article was investigated from two aspects, namely the introverted communication and the extroverted communication. Introverted communication refers to the social interactions of rural residents in the village, and its important characteristics are ceremonial interactions such as birthdays, marriages, and festivals. Such interactions often result in humanistic consumption expenditures. Therefore, this article measures introverted communication by investigating the situation of human sentiment consumption. The answer was set according to the five-level Likert scale, and the assigned value was from 1 to 5. The higher the score, the more abundant the introverted communication. Extroverted communication refers to the interactions between rural residents and outside the village. This article examines the frequency of rural residents’ outings. The more outgoings there were, the more abundant the communication network outside the village became. The options were set to “no, seldom, general, often and frequent”.

#### 3.2.3. Rural Residents’ Cognition

In this study, rural residents’ cognition includes two dimensions, namely policy cognition and responsibility awareness. Policy cognition was inspected from two aspects. One is the rural residents’ understanding of the “three-year residential environment improvement policy”, the other is the rural residents’ understanding of the environmental improvement project, which are set according to the five-level Likert scale, in which the higher the answer score is, the better the rural residents’ understanding of the policy. Responsibility awareness includes the degree of understanding, responsibility recognition, responsibility behavior, and willingness to take responsibility [55]. This article measures three aspects. The first aspect is the degree of rural residents’ recognition of the division of environmental governance responsibilities, that is, the division of responsibilities between the government and rural residents in environmental governance; the second aspect is to examine rural residents’ sense of responsibility regarding rural health governance from the behavioral perspective, that is, whether rural residents have expressed opinions to village managers on the rural health governance, and the answer is set to “no” or “yes”; the third aspect is to examine whether rural residents are willing to express their opinions to rural managers on the issue of rural health governance. The answer is set to “unwilling”, “willing”.

#### 3.2.4. Control Variables

With reference to previous studies, this article takes age, sex, and educational level as control variables. In contrast to previous studies, this model also included life pressure as a control variable to examine the impact of life pressure on rural residents’ participation in rural health governance.

### 3.3. Analytical Methods

First, this study employed statistical analysis methods to conduct a descriptive statistical analysis of rural residents’ participation, social network, and cognition in rural health governance. Secondly, SPSS 24.0 software (IBM, Armonk, NY, USA) is adopted to test the correlation between social network, rural residents’ cognition, and rural residents’ participation in rural health governance. Third, SPSS 24.0 software is used for the regression analysis of variables. In this paper, the independent variables are binary and five-category variables, and the dependent variable is a binary variable. Therefore, a binary logistic regression model was used to carry out a step-by-step regression estimation on social network, rural residents’ cognition, and participation in rural health governance, with the “enter” method selected as the specific operation approach. The reference indicators of the regression model include −2 log likelihood, Nagelkerke R, and significant results of model fitting. Finally, the fitting results of the model and the test research hypothesis are explained and examined. Since the variables of the data adopted in this paper contain missing values, the effective samples used in statistics of each step are different, and the number of effective samples used will be marked in each step.

## 4. Results

### 4.1. Descriptive Statistical Analysis

Among the 2336 valid samples, there were 1673 respondents who did not participate in rural health governance, accounting for 71.62% and 663 respondents who participated in rural health governance, accounting for 28.38%. It can be seen that the enthusiasm of rural residents to participate in rural health governance is not very high. In terms of social network, the pressure of human relationship consumption is very small, small, general, large, and very large in proportions of 2.38%, 7.30%, 39.28%, 34.36%, and 16.68%, respectively. Rural human relationship network interaction is quite abundant. The frequency of outings is not, seldom, general, often, and frequent in proportions of 5.94%, 35.82%, 33.18%, 20.11%, and 4.95%, respectively, indicating that the frequency of rural residents’ outings is not very high in 2021, which may be caused by the epidemic.

Rural residents’ cognition of rural health governance policies is the prerequisite for their participation behavior. The proportion of rural residents who have a better understanding of policy plans is 19.69%, and the proportion of rural residents who have a better understanding of remediation projects is 20.62%. The level of rural residents’ policy awareness is not high, which may be related to the fact that rural residents are busy with agricultural production. It may also be because the publicity and education work of village managers is insufficient. However, rural residents’ responsibility awareness is very high. They agree and strongly agree that rural health governance is a citizen’s responsibility, accounting for 50.09% and 35.94%, respectively, which makes up for the lack of policy cognition. At the same time, 76.09% of the interviewees expressed their willingness to express their opinions to relevant managers on rural health governance, indicating that most rural residents care about rural public affairs.

### 4.2. Correlation Analysis

Table 3 shows the relevant analysis results of rural residents’ cognition and their participation in rural health governance. In terms of related policy cognition, the Pearson chi-square test result is 0.000. With the deepening of rural residents’ understanding of residential environment governance policies and plans, their participation enthusiasm will increase from 19.32% to 63.04%. At the same time, as rural residents express their responsibilities in rural areas, their enthusiasm for participating in health and environmental governance will also increase from 20.33% to 58.79%. Through correlation analysis, it was found that there is a correlation between the cognition of rural residents and their participation in rural health governance to a certain extent, which is a typical basis for the subsequent regression model analysis, and also shows that these factors need to be incorporated into the regression model to explore the specific effect of each variable, and compare the effect size of each variable.

### 4.3. Regression Analysis of Rural Residents Participating in Rural Health Governance

Before the regression analysis, all variables were tested for multicollinearity. The results showed that the Durbin–Watson value was 1.853, which was close to 2, indicating that there was basically no autocorrelation between the variables. At the same time, the VIF value of each variable is less than 3, which meets the requirement of 0 < VIF < 10, which also shows that there is no problem of multicollinearity between variables. In order to clarify the influence of control variables, social network and rural residents’ cognition on rural residents’ participation in the rural health governance, three regression models were established using a stepwise regression method. As shown in Table 4, the control variables are incorporated into the model to produce model 1, and on this basis, social network factors are added to obtain model 2, and then rural residents’ cognition factors are added to obtain model 3. The three models have passed the significance level test (Sig. = 0.000), and the Nagelkerke R-square increased from 0.024 to 0.241, indicating that the addition of social network and rural residents’ cognitive variables can improve the fit of the model and improve the interpretation of the entire model. For social sciences, a regression model with a fit of 0.2 is qualified for analysis.

Among the control variables, sex and educational level have significant effects in Model 1 and Model 2, but not in Model 3, indicating that rural residents’ cognition can compensate for differences in sex and educational level to a certain extent. The educational level of rural residents does not have a significant impact in the three models. This is different from the research results of Marr, E.J. et al. [56]. It may be that rural health governance does not require an understanding of abstract concepts but more simple behaviors. However, life pressure is always a significant influencing factor. In Model 3, for every unit increase in life pressure, the probability of rural residents participating in the rural health governance will increase by 1.198 times.

In Model 2, the social network has a significant impact on rural residents’ participation in rural health governance. The pressure of human consumption has a positive and significant impact on rural residents’ participation in rural health governance. For every increase in pressure of human consumption by one unit, the probability of rural residents participating in rural health governance will increase by 1.271 times. This shows that the social network within the village has an impact on the behavior of rural residents. The frequency of rural residents going out has a negative and significant impact on their participation in rural health governance, that is, for every increase in the frequency of rural residents going out by one unit, their participation probability will be reduced by 0.808 times.

In Model 3, both the policy cognition and responsibility awareness of rural residents’ cognition have a significant impact on rural residents’ participation in rural health governance as well as their remediation project awareness and policy program awareness. It can be seen that responsibility perception is an important factor that affects rural residents’ participation behaviors. Compared with rural residents who have not expressed their opinions on rural health governance to village managers, the probability of participation by rural residents who have expressed their opinions will increase by 4.329 times. At the same time, the probability of participation for rural residents who expressed their willingness was 1.604 times higher than that of rural residents who did not express their willingness. With the increase in rural residents’ recognition of responsibility, the probability of their participation will also increase. For every unit of rural residents’ awareness of responsibility, the probability of their participation in rural health governance will increase by 1.453 times. Every increase in the level of rural residents’ awareness of improvement projects will increase the probability of participating in rural health governance by 1.232 times. Similarly, for every increase in the level of rural residents’ awareness of policies and programs, the probability of their participation in rural health governance will increase by 1.164 times.

## 5. Discussion

This paper constructs an analysis framework of rural residents’ social network, cognition, and participation in rural health governance, and explores the influence mechanism of rural residents’ social network and cognition on their participation behavior. The research results confirmed the hypotheses H1, H2, H3, and H4. The relationship network formed by the introverted communication of rural residents affects their participation behavior. Urbanization promotes extroverted communication among rural resident. This kind of communication weakens the emotional connection between them, and reduces their participation in rural health governance. In addition, the responsibility awareness of rural residents plays an important role in the sustainable development of rural health governance.

This study found that the greater the pressure on rural residents’ lives, the greater the probability of their participation in rural health governance. This is different from Wang M.’s research [57]. Zhifang Zhou et al. believed that the source of rural residents’ household income affects their pro-environmental behaviors, and those with high life pressures are less likely to adopt pro-environmental behaviors [58]. The normal logic of production and life is that the greater the pressure on rural residents’ lives, the more time and energy they will spend and the more they will invest in the sources of production and life rather than in public activities. A possible explanation is that rural residents use participation in rural public activities as a way to disperse life pressure and divert attention. In participating in rural public life, they can obtain support from other members and absorb external resources. At the same time, participating in public life can be a way to relieve mental stress. This may also be related to non-institutional participation in rural health governance, which is an alternative behavior of rural residents [59]. In addition, the cultural action rules formed by rural residents living in rural areas for a long time have a restrictive effect on their participation behavior, and the existing customs and norms have become the framework for understanding the logic of their actions.

Research hypotheses H1 and H2 have been confirmed: the introverted communication and the extroverted communication constitute two different types of interactions of rural residents, and there is a trade-off relationship. Introverted communication has a positive and significant effect, which is consistent with the research results of Jinhua Xie et al. [60]. Introverted communication is a communication activity carried out within a certain area. Long-term continuous ritual strengthening can accumulate social capital in rural areas, which is conducive to the holding of rural public activities and the generation of collective actions [61]. Introverted communication not only increases interactions among rural residents, but also contacts and interactions with rural area modelers, managers, and other participating members. Together, they have become the central force in rural health governance [62]. When rural areas became a half-acquaintance society, rural residents’ external exchanges increased, showing a tendency to detach themselves from the life of the rural community, and their contacts turned to urban residents or people in the same industry [40]. As rural residents increase their interactions with the outside world, they can acquire the knowledge, skills and resources they need. Rational farmers would choose “free riding” rather than contributing to rural public goods. For residents who have moved away from rural areas to settle in urban areas, it is not wise to invest time or energy in rural health governance actions.

The research hypothesis H3 confirmed that policy cognition has a significant impact on rural residents’ participation in rural health governance, which is consistent with the research results of Zheng, W. [63] and Chen, F. [64] et al. The improvement of rural residents’ cognition of policy programs and improvement projects can prompt them to engage in more reliable environmental governance behaviors [65]. The long-term increase in the level of economic income has allowed rural residents to get rid of their worries about basic living needs and begin to focus on spiritual satisfaction and improving their quality of life [66]. The cognition of the individual health part of policy cognition can promote individuals to choose hygienic individual behaviors and take actions that are beneficial to the rural public environment, such as participating in a garbage clean-up in rural areas [67]. Rural residents can incorporate the circular economy concept promoted by the government into their waste disposal behavior. The improvement of rural residents’ understanding of environmental governance policies can help them establish correct ecological and green environmental concepts, realize the dangers of environmental pollution, and agree with the policies promulgated by the government [68]. For rural managers, it can minimize environmental governance. This is the difficulty of policy implementation [69].

The research hypothesis H4 was confirmed: the awareness of responsibility has a significant impact on rural residents’ participation in rural health governance, which is consistent with the results of the study by Scott Pruysers et al. [70]. As a constituent individual of the rural unit, rural residents have certain responsibilities and obligations to the rural areas that produce and live in specific areas. When individuals recognize this responsibility and obligation, they will be shown to actively participate in rural public affairs. In other words, the awareness of responsibility will be transformed into a sense of responsibility, which in turn will shape a positive attitude of citizen participation. At the same time, the sense of responsibility will also produce pro-social behavior, reduce the cost of mobilization of rural managers, and also benefit the construction of rural resilience [71]. Rural communities are administrative units that go beyond individuals and families. The key to the success of rural collective activity is to transfer individuals’ responsibility from their family to their collective communities. Only when most rural residents put their time and energy into the construction of rural public goods can they jointly improve the effectiveness of rural health governance and achieve joint construction and sharing.

## 6. Limitations

This study has three limitations: First, the data used in this study are only from one survey, and there are no continuous follow-up survey data. The data can only reflect the current year. Secondly, there were not many analytical dimensions used in this research, and it is necessary to expand the analytical dimensions to improve the validity of interpretation. It is expected that institutional and social environmental factors will be incorporated into the analytical framework in future research. Finally, the relationship between rural residents’ social network and cognition has not been explored, and other models need to be adopted to analyze the impact of social network on rural residents’ cognition, and then on rural residents’ participation behavior, that is, to take rural residents’ cognition as an intermediary variable of social network and participation behavior.

## 7. Conclusions

Based on 2343 survey samples of Chinese rural residents, this study adopted a binary logistic regression model to explore the impact of rural residents’ social network and cognition on their participation in rural health governance. Studies have shown that social networks and cognition have a significant impact on rural residents’ participation in rural health governance, and hypotheses H1, H2, H3, and H4 were all confirmed. In the acquaintance society and the semi-acquaintance society, rural residents are involved in both introverted communication and extroverted communication. The former has a positive and significant impact on rural residents’ participation in rural health governance and the latter has a negative and significant impact. Introverted communication is rooted in kinship and geographical connections. Through ritual communication activities, social communication networks and structures are formed. It is easy to form emotional links and common interests among rural residents, while extroverted communication means that rural residents build more through industry-based interactions. The relationship network gradually deviates from the rural relationship network, and the interest relationship with the village is becoming weaker and weaker, and it is difficult to motivate them to participate in rural public life. The emergence of extroverted communication will weaken the role of introverted communication and reduce rural residents’ participation in rural health governance activities. Policy awareness can increase rural residents’ awareness of the value of environmental governance, increase policy recognition, and reduce policy implementation resistance. Responsibility awareness is the inherent driving force for rural residents to participate in rural health governance which can stimulate rural residents’ enthusiasm and initiative to participate and reduce the mobilization cost of rural managers. Based on this, by holding cultural activities, festivals, etc., rural grassroots governments can strengthen communication and exchanges between residents, enrich their social interaction activities, and increase their emotional links. Grassroots governments can also increase residents’ awareness of environmental policies and raise their awareness of environmental protection by holding specific policy training courses. It is also necessary to focus on cultivating rural residents’ sense of public responsibility and attracting more of them to participate in rural public life. For the government, it is not only necessary to pay the relevant organizational costs and training costs, but also to include this issue on the government’s agenda. Participation in sustainable health governance requires the government to develop relevant systems. For researchers, it is necessary to further analyze the influencing mechanisms of social network and modern Internet communication tools on the cognition of rural residents.

## Figures and Tables

**Table 1:** Sample characteristics.

Characteristic Index	Classification	Frequency	Proportion (%)	Standard Deviation
Sex	Male	1527	65.17	0.48
	Female	816	34.83	
Age	Below 30	109	4.65	1.14
	30–39	187	7.98	
	40–49	448	19.12	
	50–59	711	30.35	
	60 and above	888	37.90	
Occupation	Farming	1523	65.00	1.60
	Working	372	15.88	
	Teaching	29	1.24	
	Self-employed and private business owners	154	6.57	
	Rural managing	58	2.48	
	Other	207	8.83	
Marital status	Unmarried	134	5.72	0.62
	Married	1976	84.34	
	Divorced	41	1.75	
	Widowed	192	8.19	
Region	Eastern	383	16.35	0.69
	Middle	1143	48.78	
	Western	817	34.87	
In total		2343	100	

**Table 2:** Variable definitions and valuation.

Variables	Variable Name	Operational Processing	Valuation	Average	Standard Deviation
Dependent variable	Participation in rural health governance	Participate in the governance or not	No = 0; Yes = 1	0.28	0.45
Control variable	Sex	Your sex	Female = 0; Male = 1	0.65	0.48
	Age	Your age	Below 30 =1; 30–39 = 2; 40–49 = 3; 50–59 = 4; 60 and above = 5	3.89	1.14
	Educational level	Your educational level	Illiterate = 1; Primary school = 2; Junior high school = 3; High school =4; College degree or above = 5	2.64	0.95
	Life pressure	Your life pressure	No = 1; Little = 2; General = 3; High = 4; Very high = 5	3.44	0.90
Social network	Introverted communication	Human relation pressure	Very small = 1; Small = 2; General = 3; large = 4; Very large = 5	3.56	0.93
	Extraverted communication	Outgoing frequency	No = 1; Seldom = 2; General = 3; Often = 4; Frequent = 5	2.82	0.98
Residents’cognition	Policy cognition	Related policy cognition	No = 1; Little = 2; General = 3; Clear = 4; Very clear = 5	2.60	1.11
		Remediation project cognition	No = 1; Little = 2; General = 3; Clear = 4; Very clear = 5	2.66	1.05
	Responsibility awareness	Responsibility identity cognition	Very disagree = 1; Disagree = 2; General = 3; Agree = 4; Very agree = 5	4.20	0.72
		Responsibility expression	No = 0; Yes = 1	0.21	0.41
		Willingness in expression	Unwilling = 0; Willing = 1	0.76	0.43

**Table 3:** Correlation analysis between the cognition and participation in rural health governance (Unit: %, number).

Related Policy Cognition	Participation		Responsibility Expression	Participation	
	No	Yes		No	Yes
No	80.68	19.32	No	79.67	20.33
Little	77.94	22.06			
General	72.28	27.72			
Clear	59.00	41.00	Yes	41.22	58.78
Very clear	36.96	63.04			
Sample: 2334; *p* = 0.000			Sample: 2325; *p* = 0.000		

**Table 4:** Regression analysis of participation in rural health governance.

Variables	Model 1		Model 2		Model 3	
	β	Standard Error	β	Standard Error	β	Standard Error
Control variable						
Sex ^a^	0.249 *	0.101	0.282 **	0.103	0.137	0.112
Age	0.018	0.047	−0.008	0.048	−0.010	0.052
Educational level	0.162 **	0.055	0.188 ***	0.056	0.057	0.062
Life pressure	0.232 ***	0.054	0.157 **	0.057	0.181 **	0.062
Social network						
Human relation pressure			0.240 ***	0.054	0.240 ***	0.059
Outgoing frequency			−0.214 ***	0.050	−0.236 ***	0.054
Policy cognition						
Related policy cognition					0.152 *	0.077
Remediation project cognition					0.208 *	0.082
Responsibility awareness						
Responsibility identity cognition					0.374 **	0.079
Responsibility expression ^b^					1.465 **	0.119
Willingness in expression ^c^					0.472 **	0.143
constant	−2.644 ***	0.379	−2.674	0.425	−5.288 ***	0.562
Model fitting	0.000		0.000		0.000	
−2 Log Likelihood	2730.294		2672.956		2304.253	
Nagelkerke R	0.024		0.046		0.241	
Valid sample	2316		2277		2277	

Note: 1. * *p* ≤ 0.05, ** *p* ≤ 0.01, *** *p* ≤ 0.001; 2. The reference groups of “^a, b, c^” are female, no and unwilling; 3. OR = exp(β).

## Data Availability

The data presented in this study are available on request from the author (hslijie@ccnu.edu.cn). The data are not publicly available due to privacy reasons.
